# Integration of quantum key distribution and high-throughput classical communications in field-deployed multi-core fibers

**DOI:** 10.1038/s41377-025-01982-z

**Published:** 2025-08-13

**Authors:** Qi Wu, Domenico Ribezzo, Giammarco Di Sciullo, Sebastiano Cocchi, Divya Ann Shaji, Lucas Alves Zischler, Ruben Luis, Paolo Serena, Chiara Lasagni, Alberto Bononi, Tetsuya Hayashi, Alessandro Gagliano, Paolo Martelli, Alberto Gatto, Paola Parolari, Pierpaolo Boffi, Davide Bacco, Alessandro Zavatta, Yixiao Zhu, Weisheng Hu, Zhaopeng Xu, Mark Shtaif, Andrea Marotta, Fabio Graziosi, Antonio Mecozzi, Cristian Antonelli

**Affiliations:** 1 Department of Physical and Chemical Sciences, University of L’Aquila, L’Aquila, 67100 Italy; 2 Photonics Research Institute, Department of Electrical and Electronic Engineering, The Hong Kong Polytechnic University, Hong Kong, China; 3 Department of Electronic Engineering, Shanghai Jiao Tong University, Shanghai, 200240 China; 4 Department of Physics and Astronomy, University of Florence, Firenze, 50019 Italy; 5 Photonic System Laboratory, NICT, Koganei, Tokyo 184-0015 Japan; 6 Department of Engineering and Architecture, Universitá degli Studi di Parma, Parma, 43124 Italy; 7 CNIT National Laboratory of Advanced Optical Fibers for Photonics, L’Aquila, 67100 Italy; 8 Sumitomo Electric Industries Ltd., Yokohama, Kanagawa 244-8588 Japan; 9 Department of Electronics, Information and Bioengineering, Politecnico di Milano, Milano, 20133 Italy; 10 National Institute of Optics (CNR-INO), Firenze, 50125 Italy; 11 Pengcheng Laboratory, Shenzhen, 518055 China; 12 Department of Physical Electronics, Tel Aviv University, Tel Aviv, 69978 Israel

**Keywords:** Optics and photonics, Fibre optics and optical communications

## Abstract

Quantum key distribution (QKD) is a secure communication method for sharing symmetric cryptographic keys based on the principles of quantum physics. Its integration into the fiber-optic network infrastructure is important for ensuring privacy in optical communications. Multi-core fibers (MCFs), the likely building blocks of future high-capacity optical networks, offer new opportunities for such integration. Here, we experimentally demonstrate, for the first time, the coexistence of discrete-variable QKD and high-throughput classical communication in the C-band over a field-deployed MCF with industry standard cladding diameter of 125 μm. Specifically, we demonstrate successful secure-key establishment in one core of a 25.2-km uncoupled-core MCF, while simultaneously loading the remaining three cores with full C-band counter-propagating classical traffic at an aggregate net rate of 110.8 Tb/s. By proposing and experimentally validating an improved analytical model for inter-core spontaneous Raman scattering noise, we find that this configuration is optimal for our deployed MCF link as it is immune to four-wave mixing, that becomes relevant when the quantum and classical signals are propagating in the same direction. Our findings make an important step forward in demonstrating the integration of QKD and classical transmission in uncoupled-core multi-core fibers for next-generation optical communication networks.

## Abstract (web summary)

The world’s first integration of quantum key distribution and high-speed classical communications over field-deployed multi-core fiber.

## Graphical abstract

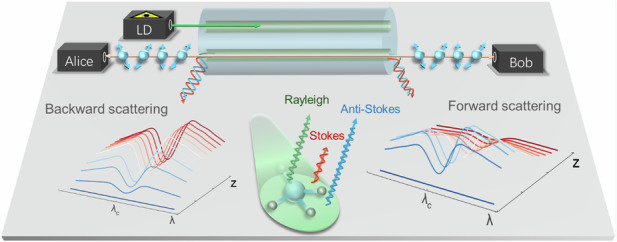

## Introduction

The protection of information privacy in communication has become an increasingly pressing challenge, and a key aspect of this challenge is ensuring the safe exchange of a cryptographic key between users. Quantum key distribution (QKD) constitutes the ultimate solution for secure key sharing, enabling remote parties to share symmetric keys with unconditional security^1–7^.

As the most prominent application of quantum communication to date, QKD stands out in being grounded in the fundamental laws of physics, contrary to other cryptographic methods, which rely on complexity. Thereby, QKD provides a level of security that is theoretically impossible to breach. Over the years, proof-of-concept experiments in single-mode fiber networks have been demonstrated in multiple countries, including the United States, Austria, Britain, Japan, Italy, China, Spain, and others, with system technologies now becoming commercial^8–16^. Nevertheless, the large-scale implementation of QKD is likely to be achievable primarily through seamless integration into the existing fiber-optic network infrastructure that has been established for classical communication systems^17–26^. The most challenging issue in integrating QKD with classical fiber optic networks is minimizing interference from classical channels transmitted in the same optical fiber^22–24^. In this context, the use of fibers supporting space-division multiplexing (SDM), such as multi-core fibers (MCFs) and multi-mode fibers, offers a unique opportunity. Such fibers have been in the spotlight of optical communications research for over a decade as a promising approach to scaling up the capacity of future fiber-optic networks^27^. In these fibers, spatial parallelism can be leveraged to separate between QKD and classical channel transmission, thereby reducing interference. This separation is particularly good in uncoupled-core MCFs^28–30^, where some cores can be dedicated to QKD while others carry classical communications. Although the inter-core coupling coefficients are typically below 60 dB/km, the residual coupling—when combined with optical nonlinear phenomena—is what limits the performance of QKD systems. To minimize this effect, the QKD channel wavelength is left unused in the cores carrying classical data. The most relevant limiting mechanism in this scenario is the phenomenon of spontaneous Raman scattering (SpRS), which is the focus of this work. Other nonlinear phenomena, such as four-wave mixing, cross-phase modulation, and cross-polarization modulation, are negligible, particularly in the counter-propagating settings on which we focus in the experimental part of this work. SpRS can occur in cores with classical signals (classical cores) and leak into the cores with QKD signals (quantum cores), or it can occur directly in the quantum cores, after the leakage of classical signals from classical cores. Therefore, in what follows, we refer to both cases as inter-core (IC) SpRS. Several pioneering studies have been performed in order to systematically investigate the impact of IC-SpRS noise on QKD performance in MCFs, while validating the feasibility of various configurations^31–34^.

However, a true proof-of-concept demonstration for the coexistence of QKD and classical transmission in MCFs must be performed with a field-deployed SDM infrastructure using MCFs that are fully loaded with classical traffic. That is because real-life environments involve a plethora of factors (e.g., temperature fluctuations, mechanical vibrations, and long-term core alignment instability) whose effect cannot be anticipated in laboratory experiments. In addition, it is crucial to use MCFs that are compatible with the 125-μm-cladding-diameter industry standard, as opposed to using MCFs with larger cladding diameters^35–41^. Adherence to this standard ensures manufacturing consistency, tooling compatibility (including compatibility with splicing and connectorization technology), and large-scale deployment^27,42^. We note that no demonstrations using fully-loaded MCFs compatible with the above-specified industry standard have been reported, even in controlled laboratory environments with spooled fibers. Finally, the coexistence of QKD and classical transmission requires optimization of core and wavelength allocation for the quantum channel. This, in turn, requires an accurate model for reliable prediction of the interference imposed by the classical channels on the QKD channel. Existing models suffer from a number of limitations that hinder accurate system design^32,34,40^. These include neglect of the frequency dependence of the relevant MCF parameters, as well as a lack of differentiation between various mechanisms contributing to IC-SpRS interference.

In this work, we successfully demonstrate the coexistence of QKD and classical transmission in a field-deployed SDM infrastructure. Our demonstration makes use of uncoupled-core four-core MCFs with a standard 125 μm-cladding diameter installed in the Italian city of L’Aquila. We demonstrate QKD using weak-coherent states with time-bin encoding and one-decoy state method, achieving a secure key rate (SKR) of 0.41 kbps over a 25.2-km MCF, while simultaneously transmitting 110.8-Tb/s full C-band wavelength-division-multiplexed (WDM) classical traffic in the three other cores. To this end, we developed a comprehensive model for the characterization of the impact of IC-SpRS. The developed model accounts for the core and frequency dependence of the relevant fiber parameters. It was experimentally validated and used to optimize the system design.

## Results

### Experimental setup of integrated optical and quantum communication architecture

**Fig. 1:**
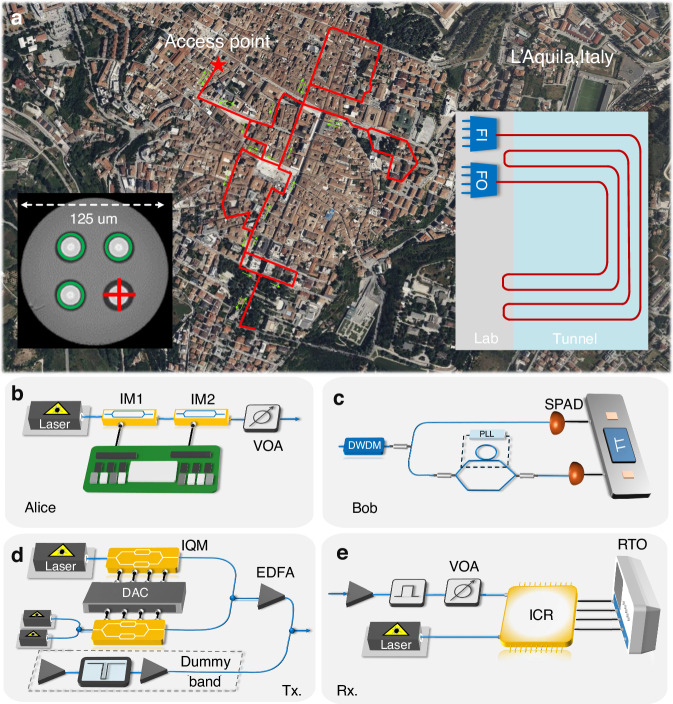
Link layout and experimental setup. **a** Map of the deployed MCF. The inset shows the cross-section image of MCF, where the + sign indicates the QKD core with the signal propagating into the fiber, while the circles indicate that the classical cores, where the signals are coming out of the fiber. The right inset shows a schematic of the fiber link and fan-in-fan-out (FIFO) devices used to address the fiber cores. The fiber link is a loop configuration and consists of four strands spliced together and to the FIFOs in the lab. **b** Quantum transmitter. **c** Quantum receiver. **d** Classical transmitter. **e** Coherent optical intradyne receiver. LD laser diode, IM intensity modulator, FPGA field programmable gate array. VOA variable optical attenuator, SPAD single photon avalanche diode, DWDM dense wavelength division multiplexing, PLL phase lock loop, TT time tagger, IQM IQ modulator, DAC digital-to-analog converter, EDFA erbium-doped fiber amplifier, ICR intradyne coherent receiver, RTO real-time oscilloscope

We conducted this co-existence experiment using a field-deployed MCF testbed located in the Italian city of L’Aquila^43^. The key parameters of the deployed MCF, including attenuation, crosstalk coefficients, and Raman efficiency, were experimentally characterized and are detailed in the Supplementary Material. The experimental setup is depicted in Fig. 1, which also includes a map illustrating the deployed MCFs. As illustrated in the cross-sectional image of the MCF, the cross symbol denotes the quantum signal entering the fiber core, while the three green circles represent the counter-propagating classical signals in the adjacent and diagonal cores. Alice and Bob employed a three-state efficient BB84 protocol with time-bin encoding and a one-decoy-state approach. A comprehensive description of the protocol is provided in the Methods section. The protocol utilizes quantum states made by coherent state attenuated down to single-photon level. The optical intensity corresponds to a mean photon number per pulse of *μ*_1_ or *μ*_2_, consistent with the decoy-state method^44–47^. These states are encoded in one of two mutually unbiased bases, *Z* or *X*, with corresponding probabilities *P*_Z_ = 50% and *P*_X_ = 50%. The quantum transmitter shown in Fig. 1b is based on attenuated coherent states. A beam generated by a continuous-wave C-band laser at 1538.19 nm passed through an intensity modulator driven by an electrical signal coming from a field-programmable gate array (FPGA). The sequence of quantum states was generated based on a pseudo-random binary sequence of length 4095, repeated 145,358 times per second, for a final state generation rate of 595.241 MHz. At the output, the optical signal encoded the desired sequence of quantum states in a time-bin format, and a second intensity modulator adjusted the intensity of certain states according to the basis and the requirements of the one-decoy state method. Finally, a variable optical attenuator reduced the intensity to the single-photon level per pulse. The exact intensity, expressed as the average photon number per pulse, was optimized to maximize the SKR in a simulation model accounting for all the channel and receiver characteristics. The optimal values of *μ*_1_ and *μ*_2_ were determined to be *μ*_1_ = {0.06, 0.08, 0.1, 0.12} and *μ*_2_ = {0.03, 0.04, 0.05, 0.06} for the distance of 6.3 km, 12.6 km, 18.9 km, and 25.2 km, respectively. The average number of photons per pulse is lower for shorter distances to account for the saturation of single-photon detectors.

In Fig. 1c, at Bob’s end, a 100-GHz dense wavelength division multiplexing (DWDM) filter was used for the quantum core after the fan-in and fan-out (FIFO) device to remove the out-of-band Raman noise, with an isolation of ~50 dB between channels. The quantum receiver consisted of a beam splitter that acted as a basis selector, after which some photons directly impinged upon a single-photon avalanche diode (SPAD) for Z basis measurement. The X basis measurement required to read the phase between two pulses, so the states first passed through a Mach-Zehnder interferometer with a delay of 800 ps between the two arms, the separation between the two time bins. Another laser, with a slightly different wavelength, was passed through the interferometer in the counter-propagating direction. Its power was monitored and used as feedback for a proportional-integral-derivative controller that acted on a phase shifter. This system behaved as a phase-lock loop (PLL) that stabilized the interferometer. One output of the interferometer was connected to a second SPAD to measure $\left\vert -\right\rangle$ states and assess the quantum bit error rate. Additional DWDM filters were used to separate the PLL laser and improve the filtering of the quantum signal. The entire QKD system was fully implemented in fiber. In this co-existence experiment, the quantum laser was tuned to a wavelength of 1538.19 nm. The use of this specific wavelength resulted from the constraints of the available DWDM filter.

In the classical transmission taking place in the three classical cores, full C-band loading was implemented by means of one test channel for performance evaluation and broadband noise. The transmitter and receiver are illustrated in Fig. 1d, e. The test channel was a 20-GBd dual-polarization (DP) 256-ary quadrature-amplitude modulated signal generated using 100-GSa/s digital-to-analog converters, a DP-IQ modulator, and a tunable laser. For the dummy band implementation, we employed Erbium-Doped Fiber Amplifiers (EDFAs) and an optical processor to generate amplified spontaneous emission (ASE) noise with a flattened spectrum, incorporating a 100-GHz notch at 1538.19 nm (corresponding to the quantum-channel wavelength) and a notch at the tunable laser’s wavelength to accommodate the test signal and its two neighboring channels. The modulated optical signal and flattened ASE noise were then combined and pre-amplified by an EDFA. To characterize the WDM channel performance, we swept the tunable laser wavelength across the entire C-band while simultaneously adjusting the optical processor’s frequency response accordingly. The combined signal was subsequently divided by a 1 × 4 splitter, with three of the four outputs injected into the fan-in at a launch power of 19.5 dBm per core. At the receiver side, a standard intradyne coherent receiver was utilized for classical signal detection. It should be noted that the wavelength sweeping process inevitably induced transient optical instabilities at the quantum channel wavelength, implying long waiting times for the QKD system to stabilize prior to each measurement. This was avoided by adopting a two-step approach: first, we assessed the QKD performance using a dummy band encompassing the entire C-band, with the exception of the quantum channel; subsequently, we conducted separate measurements of classical data rates at the same predetermined power level with the quantum receiver disconnected from the fiber. The main justification of this approach is that the performance of classical data transmission is not affected to any extent by the quantum channel, whose power is negligibly low by design. Furthermore, performing classical data rate measurement while running the QKD system would have exposed it to the risk of damage, owing to transient optical instabilities caused by the wavelength sweeping process. Note that the need for wavelength sweeping and the instabilities that it causes are absent in real commercial systems.

### Quantum key and classical data rates

**Fig. 2:**
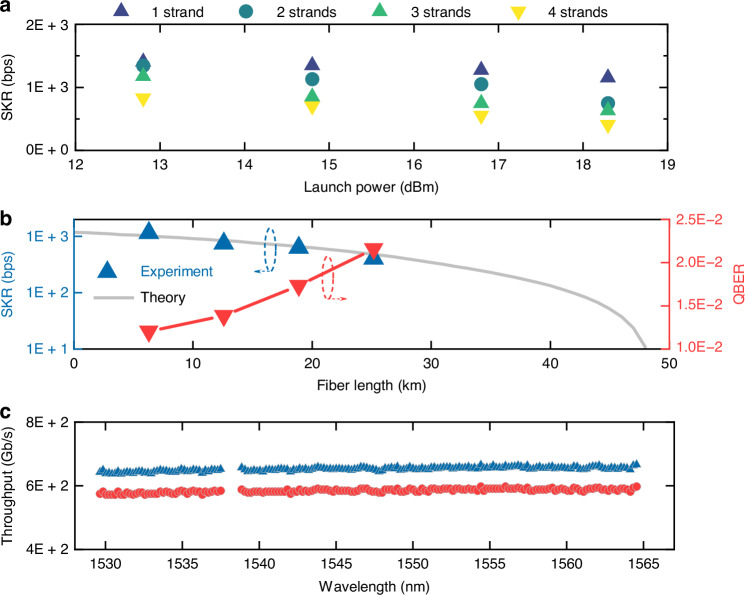
Experimental results. **a** SKR versus launch power per fiber core. **b** SKR and QBER versus fiber length. Blue triangles: measured SKRs. Red triangles: measured QBERs in one fiber core as a function of fiber length, with counter-propagating classical signals at 18.3 dBm launched in the remaining three cores. Gray line: theoretical prediction. **c** Spatially aggregated throughput of 3 × 170 SDM and WDM classical channels. Triangles: generalized mutual information. Circle: decoded throughput

To quantitatively assess the impact of SpRS noise from the classical signals on the SKR, we developed an analytical model, as described in the “Methods” section. Our model offers two key advancements over previously reported approaches^32,34,40^. First, it is built upon the accumulated electric-field formulation of the distributed Raman effect, rather than upon a power-based description. This approach provides a more accurate and physically consistent representation of the SpRS process, thereby improving the precision of QKD performance predictions. Second, our model incorporates core- and wavelength-dependent parameters, including fiber loss, inter-core coupling coefficients, and Raman efficiency, enabling optimal core and wavelength allocation for quantum signals. This enables using experimental measurements of these fiber parameters. The model allows to estimate the SpRS-induced photon count rate on SPADs (as discussed in the next section and “Methods”) and assess its impact on SKR. The analysis demonstrates that counter-propagation exhibits lower Raman noise power in fiber links shorter than 50 km, whereas co-propagation becomes advantageous for longer distances. Based on these findings, we experimentally validated the optimal configuration by transmitting classical signals in the counter-propagating configuration. Figure 2a shows the SKR as a function of the launch power into each classical core after the fan-in device for different link lengths. These lengths were obtained by concatenating one to four fiber strands, each measuring 6.3 km. For a single fiber strand, the SKR decreases from 1.4 to 1.15 kbps as the launch power increases from 12.8 to 18.3 dBm. This decreasing trend persists across multiple fiber strands. As expected, the QKD performance deteriorates slightly with the power of the classical channels.

Next, we injected an optical signal with a power of 19.5 dBm (18.3 dBm after the fan-in) into each of the three classical cores, counter-propagating with respect to the quantum signal. We tested four different channel attenuation values, corresponding to transmission over one, two, three, and four fiber strands. Figure 2b shows the SKR and quantum bit error rate (QBER) measured at different link lengths. As the link length increases, the SKR decreases from 1.15 to 0.41 kbps, while the QBER increases from 0.012 to 0.021. This follows from a corresponding increase in link loss and Raman noise power. The theoretical curve in the figure was obtained by reproducing the experimental conditions in simulation. This required accounting for additional implementation-related impairments, such as the insertion loss of the DWDM filter, the loss introduced by the FIFO components, and the loss due to imperfect splices and connectors.

The results in Fig. 2b validate the reliability of the proposed model in analyzing the impact of Raman noise and predicting the performance of the QKD system. We measured the classical data rates for the three classical cores at a launch power of 19.5 dBm. The total throughput per wavelength is plotted in Fig. 2c in terms of generalized mutual information (triangles) and decoded throughput (circles). Negligible wavelength dependence of the measured data rates can be observed. The system achieves a decoded throughput of 99.3 Tb/s, with an estimated achievable information rate of 110.8 Tb/s based on generalized mutual information.

Table 1 presents a comparison between this work and state-of-the-art discrete-variable QKD implementations co-existing with classical communication over MCFs. The comparison relates to the QKD protocol, decoy-state method, SKR, number of classical channels, total throughput, MCF diameter, transmission distance, and demonstration environment. Notably, our system demonstrates coexistence with the highest classical throughput to date—110.8 Tb/s—using 510 (170 × 3) WD × SDM channels over a field-deployed MCF with an industry-standard 125-μm cladding diameter. We note that the main limitation to the SKR in our experiment was imposed by the use of regular optical-communication equipment lacking thermal and mechanical stabilization. As shown in Fig. 2a, a moderate reduction in SKR, ranging between ~15% (for a single fiber strand) and 50% (for four fiber strands), is observed by increasing the classical transmission power by 6 dB. At any rate, it is important to stress that the nominal SKR value is immaterial to this work’s implications regarding the coexistence between QKD and classical transmission. Our results mark an important step towards the deployment of quantum-classical coexisting systems.

**Table 1:** Comparison of state-of-the-art QKD-MCF experiments co-existing with classical communication

Ref.	^35^	^36^	^37^	^38^	^40^	^41^	This work
QKD protocol	BB84 (T12)	3-state BB84	BB84 (phase)	BB84	BB84	BB84	3-state BB84
Decoy-state method	Two	One	One	N/A	Two	One	One
Detecting technology	Self-diff. SPAD	SNSPD	SPAD	SPAD	SPAD	SPAD	SPAD
SKR (bps)	605 k	2.86 M	10.9 k	1.4 k	16 k	4.4 k	1.15 k/0.41 k
# Classical channels (WDM × SDM)	2 × 5	1 × 37	N/A	8 × 6	N/A	1 × 6	170 × 3
Throughput (Tb/s)	0.1	0.37	N/A	9.6	N/A	0.67	110.8
MCF Diameter (μm)	185	248	>150	>180	>150	>150	125
# Cores	7	37	7	7	7	7	4
Distance (km)	53	7.9	1	1	10	2.5	6.3/25.2
Overall loss (dB)	14.1	3.75	5.9	6.74	-	>0.5	6.8–17.9
Environment	Lab	Lab	Lab	Lab	Lab	Lab	**Field**

### Experimental validation of spontaneous Raman scattering noise model

**Fig. 3:**
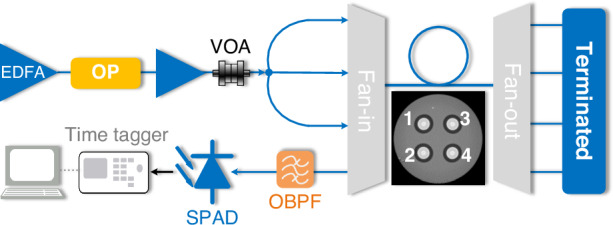
Experimental setup to measure the photon counts of SpRS noise. OP optical processor, SPAD single-photon avalanche diode, OBPF optical band-pass filter

**Fig. 4:**
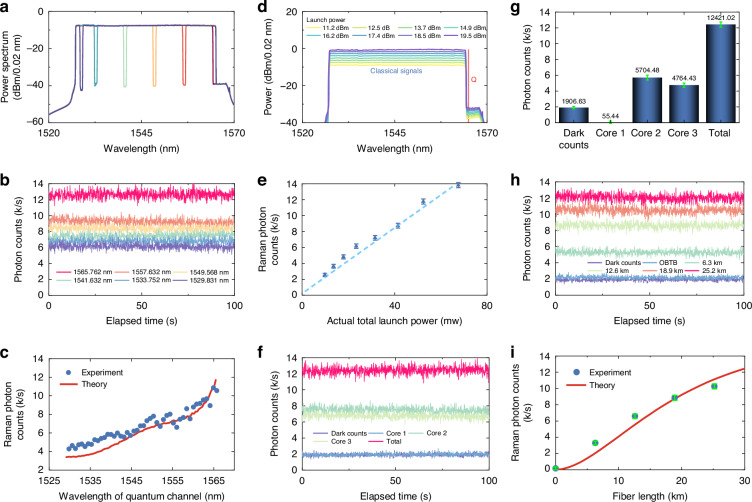
Measurement of SpRS noise. **a** Optical spectra with different notches. **b** Measured photon counts over time when the quantum receiver is at different notches (one active at a time). **c** Raman photon counts as a function of the quantum channel’s wavelength. The solid line is the theory. **d** Optical spectra with different launch powers to the fiber core. The quantum channel (Q) is at the longest wavelength in this and subsequent panels. **e** Raman photon counts versus launch power. **f** Measured photon counts over time from cores 1–3 and all cores. **g** Contributions of dark counts, cores 1–3, and all cores. **h** Measured photon counts over time at different fiber lengths. **i** Raman photon counts as a function of the fiber length. The solid line is the theory

SpRS noise photons may arrive within the observed time window and be mistakenly registered as quantum signal detections, thereby increasing the QBER and decreasing the SKR, even if the dark count level of the single-photon detector remains unchanged. To further validate the integration of quantum and classical optical communications in MCFs, we designed and conducted a photon-counting experiment with the goal of quantitatively characterizing the SpRS noise. As shown in Fig. 3, an optical processor was employed to spectrally flatten ASE noise, simulating a fully-loaded C-band signal. Within this flattened spectrum, a 100-GHz notch was carved at the quantum wavelength. Following noise shaping, the signal was further amplified to achieve a practical launch power of ~20 dBm per core, consistent with fully-loaded C-band operation. The flattened ASE noise was split into three copies and injected into cores 1, 2, and 3 via a FIFO device. Spectra with notches at various wavelengths are shown in Fig. 4a. Notably, since we are emulating classical channels with broadband noise, some noise photons are present in the notch as a result of imperfect filtering. This issue does not exist in commercial systems when no classical signals are transmitted within the QKD channel bandwidth. In the co-propagating transmission scheme, this residual noise at the quantum wavelength may easily become dominant over SpRS, and this seemed to be the case in our experiment. Therefore, we focused on the counter-propagating scheme to measure the power of backward SpRS noise. Here, the residual noise in the notch propagates backward only after Rayleigh back-scattering in the quantum core, which implies sufficient additional suppression to make it negligible.

The receiver was connected to the quantum core (core 4 in Fig. 3) to measure the photon counts of backward Raman noise. The receiver setup included a tunable optical filter with a bandwidth of 0.2 nm, a SPAD (ID221, IDQuantique SA) with a 20% detection efficiency and a 20-μs dead time, and a time tagger (Time Tagger 20 by Swabian Instruments) for photon counting. Photon counts were measured and calibrated, considering the saturation effect of the SPAD. The MCF used in the experiment consisted of four strands of deployed uncoupled-core 4-core fiber of 6.3 km each^43^. The FIFO device introduced an insertion loss of ~2.4 dB (1.2 dB for fan-in and 1.2 dB for fan-out).

First, we investigated the photon counts as a function of wavelength to determine the optimal allocation for the quantum channel. The quantum channel (notch channel) was swept across wavelengths from 1529.831 to 1565.762 nm, with a channel spacing of 100 GHz. The ASE-emulated C-band signal was launched at a power of 19.5 dBm per core. Figure 4b illustrates the measured photon count rate for six typical wavelength allocations of the notch channel over a 100-s observation period. Lower photon counts were measured at shorter wavelengths, as expected. We measured the photon counts across all quantum-channel wavelengths and the results are plotted in Fig. 4c. We note that a dark count rate of 1.9 kHz was subtracted in all cases to isolate the Raman photon counts. The solid curve illustrates the theoretical predictions based on the proposed model, incorporating an effective OBPF bandwidth of 0.24 nm and accounting for a total insertion loss of 5.7 dB arising from the OBPF and fan-in components. Consistent with theory—the photon counts increase with increasing wavelengths due to the higher Raman scattering efficiency at Stokes (longer) wavelengths relative to the pump, confirming that shorter wavelengths are indeed preferable for quantum channel allocation to mitigate the impact of SpRS noise. The slight discrepancies between the experimental results and theoretical predictions can be attributed to the inherently random and time-varying nature of inter-core coupling.

Next, we injected the C-band signal at varying launch powers, ranging from 11.2 to 19.5 dBm, to analyze the impact of classical signal power. The optical spectra with different launch powers are shown in Fig. 4d. For this measurement, we assigned the longest wavelength to the quantum channel to maximize the Raman photon counts and minimize the influence of dark counts on the results. The Raman photon counts, after removal of the dark counts, are plotted in Fig. 4e as a function of the launch power (in mW), taking into account the 1.2 dB insertion loss from fan-in. The results demonstrate the expected linear increase in Raman photon counts with launch power. When no classical signals are injected into the fiber, only dark counts are present, resulting in Raman photon counts approaching zero. The linear increase of the photon counts with launch power is also an indication of the fact that this transmission scheme is not affected by four-wave mixing (which would imply a growth in photon counting proportional to the third power of the launch power).

The individual contributions of cores 1, 2, and 3 to the photon counts from core 4 (i.e., by loading only the corresponding core), as well as their aggregate contribution, were measured and are presented in Fig. 4f. The results indicate that the contribution of the diagonal core is indistinguishable from dark counts, suggesting that there is negligible backward Raman noise from the diagonal core. The Raman photon counts from the individual cores, after subtracting dark counts, are shown in Fig. 4g. The photon counts from cores 2 and 3 are significantly higher than those from core 1. We note that the sum of the individual photon counts, including contributions from all cores and dark counts, matches well the measurements obtained when all cores are excited.

Finally, we examined the impact of fiber length on backward Raman photon counts. The measurements were taken in the optical back-to-back configuration and with 1, 2, 3, and 4 spans of 6.3-km MCF. The results are shown in Fig. 4h. They indicate that backward Raman photon counts increase with fiber length. After dark counts are subtracted, the Raman photon counts exhibit a trend of approximately linear growth at short fiber lengths, eventually saturating as the fiber length increases. This is because of the increase of the attenuation affecting the propagation of both the classical signal and the SpRS noise, so that the noise generated close to the QKD receiver dominates the overall noise power. A plot of the Raman photon counts versus fiber length is shown in Fig. 4i, where the solid curve is the theory, clearly in good agreement with the measurements.

## Discussion

We successfully demonstrated a three-state efficient BB84 protocol using time-bin encoding and the one-decoy state method within a field-deployed MCF testbed in L’Aquila, Italy. To this end we developed and experimentally validated a comprehensive analytical model for the accumulation of SpRS noise. By accounting for the core and frequency dependence of the fiber parameters, and by properly including the mechanisms that produce SpRS, the model achieved high accuracy and was used for system optimization. Specifically, SKRs of 1.15 kbps, 0.75 kbps, 0.63 kbps, and 0.41 kbps were achieved over MCF link lengths of 6.3 km, 12.6 km, 18.9 km, and 25.2 km, respectively, while transmitting classical traffic at 110.8 Tb/s. Our measurements, including Raman photon counts, corroborate the accuracy of the SKR predictions obtained with the model. Inter-core SpRS noise generated by classical signals and its impact on QKD were systematically studied in the context of coexisting classical and quantum transmission in uncoupled-core MCFs. We investigated multiple transmission schemes in which the QKD signal is assigned to a specific wavelength within a dedicated core, while the remaining wavelengths across the other cores are utilized for classical data transmission. The model relates to two mechanisms of Raman-induced interference. One, where the classical signal couples into the quantum core and generates SpRS therein, and another, where SpRS is generated in the classical cores and then couples into the quantum core.

Our study reveals three key results. First, in all scenarios, allocating the quantum channel to the short-wavelength side of the C-band is preferable due to the reduced SpRS noise. Second, contrary to previous studies^32^, we demonstrated (as detailed in the “Methods” section) that the two above-described mechanisms have different dependencies on fiber parameters and may have different weights. Third, only the cores adjacent to the quantum-carrying core significantly affect QKD performance. The diagonal core exerts negligible influence on the quantum signal, owing to the increased core pitch between diagonal cores, which leads to a very weak coupling with the quantum core, measured to be less than 70 dB/km. We also showed that counter-propagation produces reduced Raman noise power in fiber links below 50 km in length, while co-propagation is preferable for longer-distance transmissions. However, it must be pointed out that this conclusion may be offset somewhat by accounting for the effect of four-wave mixing, which is present only in the co-propagating schemes—an aspect that is being addressed in a separate endeavor.

According to theoretical predictions in Fig. 2b, secure key extraction remains feasible over a transmission distance of up to 48 km in one fiber core, even when the other three cores are utilized for full C-band DWDM transmission. At this distance, classical communication is also reliably achieved with no data rate degradation, suggesting that the integration of quantum and classical communication over MCF can be further extended to secure metropolitan area networks.

Overall, MCFs provide a promising platform for supporting not only discrete-variable QKD protocols but also more advanced schemes such as measurement-device-independent QKD and continuous-variable QKD^4,48^. These protocols are particularly advantageous in enhancing security and enabling seamless integration with existing classical communication infrastructures. These advanced QKD protocols can further benefit from the spatial multiplexing and isolation characteristics of MCFs, which help improve the SKR, robustness, and scalability of quantum-classical co-transmission systems.

## Materials and methods

### Three-state efficient BB84 protocol with time-bin encoding and one-decoy-state method

The protocol adopted in this work is the three-state efficient BB84 with time-bin encoding and the one-decoy state method^44–47^. This protocol is designed such that one basis is used for key encoding, while the other is solely dedicated to security checks. In this scheme, Alice prepares the states $\left\vert 0\right\rangle$ and $\left\vert 1\right\rangle$ in the computational basis (or Z basis) and only the state $\left\vert +\right\rangle$ in its mutual unbiased basis, the X basis. Bob performs conventional measurements in the Z basis and, when measuring in the X basis, projects the quantum states onto $\left\vert -\right\rangle$, orthogonal to $\left\vert +\right\rangle$. As time-bin and phase encoding is adopted, each time frame corresponding to a quantum state consists of two time bins, typically referred to as early (*e*) and late (*l*). A photon arriving in the first time bin corresponds to the quantum state $\left\vert 0\right\rangle ={\left\vert 1\right\rangle }_{e}{\left\vert 0\right\rangle }_{l}$, while a click in the second time bin determines the state $\left\vert 1\right\rangle ={\left\vert 0\right\rangle }_{e}{\left\vert 1\right\rangle }_{l}$. The superposition basis involves both pulses; in the single-photon regime, this can be viewed as a single photon whose wave function spreads across the two time bins. In this context, $\left\vert +\right\rangle =\frac{1}{\sqrt{2}}({\left\vert 0\right\rangle }_{e}{\left\vert 1\right\rangle }_{l}+{\left\vert 1\right\rangle }_{e}{\left\vert 0\right\rangle }_{l})$ and $\left\vert -\right\rangle =\frac{1}{\sqrt{2}}({\left\vert 0\right\rangle }_{e}{\left\vert 1\right\rangle }_{l}-{\left\vert 1\right\rangle }_{e}{\left\vert 0\right\rangle }_{l})$. In the Z basis the states are trivially decoded by observing the photon’s time of arrival in a single-photon detector. Measurement in the X basis instead requires determining the relative phase between the $\left\vert 0\right\rangle$ and $\left\vert 1\right\rangle$ components. This can be done by using a delay interferometer to measure the interference between the two time bins. The requirement of measuring only the $\left\vert +\right\rangle$ state reduces the complexity of both quantum transmitter and receiver. By implementing the efficient version, in which the probability of generating and measuring photons is not equal in the two bases, this protocol also outperforms standard BB84 in terms of key exchange rate.

This protocol’s security has been proven in the context of finite-size key and decoy-state methods^49^. Here, the key length *l*_key_ is bound to

1 $${l}_{{\rm{key}}}\le {s}_{z,0}^{l}+{s}_{z,1}^{l}\left[1-{H}_{2}({\phi }_{z}^{u})\right]-{\lambda }_{{\rm{EC}}}-6\,{\log }_{2}\left(\frac{19}{{\varepsilon }_{\sec }}\right)-{\log }_{2}\left(\frac{2}{{\varepsilon }_{{\rm{corr}}}}\right)$$

where ${s}_{Z,0}^{l}$ and ${s}_{Z,1}^{l}$ are the lower bounds for the vacuum and single-photon events, respectively, ${\phi }_{z}^{u}$ is the upper bound of the phase error rate, *λ*_EC_ is the number of disclosed bits during the error correction stage, ${H}_{2}(x)=-x\,{\log }_{2}(x)-(1-x)\,{\log }_{2}(1-x)$ is the Shannon entropy function for a binary variable, and ${\varepsilon }_{\sec }$ and *ε*_corr_ are the secrecy and correctness parameters. The last two terms are the price to pay to ensure that the key is secret and correct up to a probability of ${\varepsilon }_{\sec }+{\varepsilon }_{{\rm{corr}}}$. Post-processing is performed on blocks of 10^7^ bits, with the secrecy and correctness coefficients set to ${\varepsilon }_{\sec }={\varepsilon }_{{\rm{corr}}}=1{0}^{-12}$.

### Model for distributed Raman scattering in uncoupled-core multi-core fibers

**Fig. 5:**
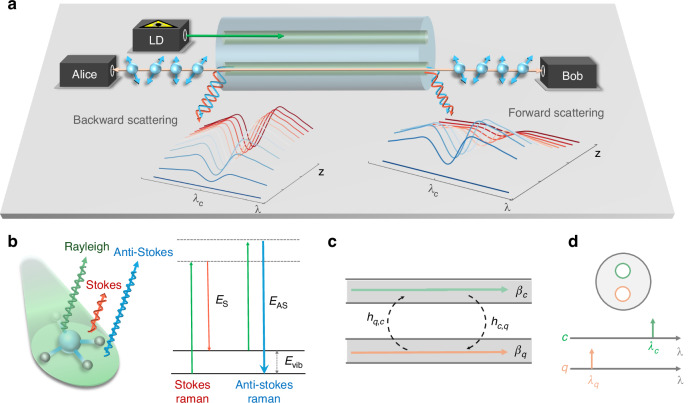
Scheme for the integration of QKD and classical communications in MCFs. **a** Schematic diagram of the integration of classical and quantum communications. LD laser diode. Two-core configuration is shown for the sake of illustration. The insets of backward and forward scattering show the Raman noise power as a function of wavelength and fiber length when the classical signal is at *λ*_*c*_. **b** Raman scattering process. **c** Inter-core coupling. **d** Wavelength and core configuration for quantum and classical signals. Green represents classical signals, while orange denotes quantum signals

The schematic diagram of simultaneous classical communication and quantum communication is shown in Fig. 5a. For clarity of illustration, we assume a two-core fiber configuration. The classical signal is assigned to core *c*, at wavelength *λ*_*c*_, while the quantum signal is transmitted at a different wavelength *λ*_*q*_ in core *q*. In what follows, we refer to these wavelengths as the *c*-th and the *q*-th wavelengths. The wavelength and core configurations are detailed in Fig. 5d. We consider each parameter to be wavelength- and core-dependent, including attenuation *α*_*c*_(*λ*) and *α*_*q*_(*λ*), linear power coupling coefficients *h*_*c*,*q*_(*λ*), and Raman efficiencies, which quantify the conversion efficiency of the Raman scattering process where an incident photon interacts with a phonon, thereby generating Raman-scattered light, *η*_*c*_(*λ*_*c*_, *λ*_*q*_) and *η*_*q*_(*λ*_*c*_, *λ*_*q*_).

The main source of noise impairing quantum communications in fiber channels, where quantum signals coexist with classical signals is SpRS originating from the classical signals. In the case of MCFs, where quantum and classical signals are also spatially separated, the SpRS noise reaches the quantum channel via inter-core crosstalk. In what follows, we elaborate on a simple model for the two propagation phenomena. SpRS is illustrated in Fig. 5b. In this process, an incident photon from the classical signal interacts with an optical phonon, temporarily exciting it to a virtual energy state. This interaction results in scattered photons with altered energy (and consequently, different wavelengths). This scattering can produce two possible outcomes: (i) Stokes scattering: energy is transferred from the photon to the phonon and hence the scattered photon has a longer wavelength than the incident photon; (ii) Anti-Stokes scattering: energy is transferred from the phonon to the photon and hence the scattered photon has a shorter wavelength than the incident photon. Moreover, SpRS occurs in both the same propagation direction of the classical signal that produces it and in the opposite direction. The relevant component is the one traveling in the same propagation direction as the quantum signal. Here, we refer to the propagation direction of the classical signal in core *c* as the forward propagation direction, extending from *z* = 0 to *z* = *L*, where *z* = 0 is the fiber input and *L* is the fiber length. Therefore, the terms backward propagation direction and counter propagation refer to the opposite propagation direction, extending from *z* = *L* to *z* = 0.

The forward propagating SpRS field at the quantum wavelength *E*_SpRS_(*z*) accumulates according to the following evolution equation

2 $$\begin{array}{l}{\rm{d}}{E}_{{\rm{SpRS}}}(z)=-\frac{{\alpha }_{k}({\lambda }_{q})}{2}{E}_{{\rm{SpRS}}}(z){\rm{d}}z+{\rm{d}}N(z),\\\quad\quad\quad\quad\quad k\in (c,q)\end{array}$$

where the complex field d*N*(*z*) represents the noise contribution generated by the classical signal within the fiber segment d*z*, with the initial field *E*_SpRS_(0) = 0 (here and in some of the subsequent expressions, we omit the wavelength dependence for ease of notation). Note that the term describing stimulated Raman scattering (that always accompanies spontaneous emission) is omitted, owing to low power of the classical signal acting as a pump, which makes the net Raman gain *G*_*R*_−1 across the fiber link much smaller than unity. Therefore, the field of the SpRS noise is given by

3 $${E}_{{\rm{SpRS}}}(z)=\mathop{\int}\nolimits_{0}^{z}{e}^{-\displaystyle\frac{{\alpha }_{k}({\lambda }_{q})}{2}(z-{z}^{{\prime} })}{\rm{d}}N({z}^{{\prime} })$$

and its power spectral density (PSD), to which in what follows we refer simply as Raman noise power, can be calculated as

4 $${P}_{{\rm{SpRS}}}(z)=\left\langle | {E}_{{\rm{SpRS}}}(z){| }^{2}\right\rangle =\mathop{\int}\nolimits_{0}^{z}\mathop{\int}\nolimits_{0}^{z}{e}^{-\displaystyle\frac{{\alpha }_{k}({\lambda }_{q})}{2}(z-{z}^{{\prime} })}{e}^{-\displaystyle\frac{{\alpha }_{k}({\lambda }_{q})}{2}(z-{z}^{{\prime\prime} })}\left\langle {\rm{d}}N({z}^{{\prime} }){\rm{d}}{N}^{* }({z}^{{\prime\prime} })\right\rangle$$

The power of the Raman noise contribution d*N* is proportional to the propagating classical signal power, which in this framework we refer to as pump signal with power *P*_*p*_(*z*), with the proportionality coefficient being the Raman efficiency of the core where the Raman effect occurs *η*_*k*_(*λ*_*c*_, *λ*_*q*_), and is spatially uncorrelated, namely

5 $$\langle {\rm{d}}N({z}^{{\prime} }){\rm{d}}{N}^{* }({z}^{{\prime\prime} })\rangle ={\eta }_{k}({\lambda }_{c},{\lambda }_{q}){P}_{p}({z}^{{\prime} })\delta ({z}^{{\prime} }-{z}^{{\prime\prime} }){\rm{d}}{z}^{{\prime} }{\rm{d}}{z}^{{\prime\prime} }$$

with the result

6 $${P}_{{\rm{SpRS}}}(z)=\mathop{\int}\nolimits_{0}^{z}{e}^{-{\alpha }_{k}({\lambda }_{q})(z-{z}^{{\prime} })}{\eta }_{k}({\lambda }_{c},{\lambda }_{q}){P}_{p}({z}^{{\prime} }){\rm{d}}{z}^{{\prime} }$$

With similar arguments, one can find that the backward-scatter Raman noise power is given by

7 $${P}_{{\rm{SpRS}},{\rm{B}}}(z)=\mathop{\int}\nolimits_{z}^{L}{e}^{-{\alpha }_{k}({\lambda }_{q})({z}^{{\prime} }-z)}{\eta }_{k}({\lambda }_{c},{\lambda }_{q}){P}_{p}({z}^{{\prime} }){\rm{d}}{z}^{{\prime} }$$

Note that Eqs. (6) and (7) can be used to describe both the accumulation of SpRS in the classical core, where the pump is the information-carrying classical signal (*k* = *c*), and in the quantum core (*k* = *q*), where the pump is the result of the cross-talk from the classical to the quantum core. In each case the pump is at the *λ*_*c*_-th wavelength and the Raman field at the *λ*_*q*_-th wavelength.

For the description of inter-core cross-talk, we adopt a power-coupled-equation-based model. Denoting the *z*-dependent signal powers in the classical and quantum cores by *P*_*c*_ and *P*_*q*_, respectively, the power coupling equations can be expressed as

8 $$\frac{d{P}_{c}}{dz}=-{\alpha }_{c}{P}_{c}-{h}_{c,q}{P}_{c}+{h}_{q,c}{P}_{q}$$

9 $$\frac{d{P}_{q}}{dz}=-{\alpha }_{q}{P}_{q}-{h}_{q,c}{P}_{q}+{h}_{c,q}{P}_{c}$$

where *h*_*c*,*q*_ = *h*_*q*,*c*_ represents the power coupling coefficient between the two cores derived in ref. ^50^. The second terms on the right-hand side of Eqs. (8) and (9) can be neglected, because their effect is equivalent to increasing the loss coefficients by *h*_*c*,*q*_ << *α*_*c*,*q*_. We also neglect the third term on the right-hand side of Eq. (8), because the coupling is weak and therefore *P*_*c*_ >> *P*_*q*_ (i.e., the undepleted-pump approximation). As a result, the power-coupled equations reduce to the following form,

10 $$\frac{d{P}_{c}}{dz}=-{\alpha }_{c}{P}_{c}$$

11 $$\frac{d{P}_{q}}{dz}=-{\alpha }_{q}{P}_{q}+{h}_{c,q}{P}_{c}$$

At the classical wavelength, under the assumption of *z*-independent coupling coefficients, the solution of Eqs. (10) and (11) for *P*_*c*_(0, *λ*_*c*_) = *P*_0_ and *P*_*q*_(0, *λ*_*c*_) = 0 reads

12 $${P}_{c}(z,{\lambda }_{c})={P}_{0}{e}^{-{\alpha }_{c}({\lambda }_{c})z}$$

13 $${P}_{q}(z,{\lambda }_{c})={P}_{0}{h}_{c,q}({\lambda }_{c})\frac{{e}^{-{\alpha }_{q}({\lambda }_{c})z}-{e}^{-{\alpha }_{c}({\lambda }_{c})z}}{{\alpha }_{c}({\lambda }_{c})-{\alpha }_{q}({\lambda }_{c})}$$

At the quantum wavelength, the following general solution should be used,

14 $${P}_{q}(z,{\lambda }_{q})={h}_{c,q}({\lambda }_{q})\mathop{\int}\nolimits_{0}^{z}{e}^{-{\alpha }_{q}({\lambda }_{q})(z-{z}^{{\prime} })}{P}_{c}({z}^{{\prime} },{\lambda }_{q})d{z}^{{\prime} }$$

where ${P}_{c}({z}^{{\prime} },{\lambda }_{q})$ is the quantum signal propagating in the same propagation direction as the classical signal. In the case of a backward propagating quantum signal, the above can be readily adapted with the following result

15 $${P}_{q}(z,{\lambda }_{q})={h}_{c,q}({\lambda }_{q})\mathop{\int}\nolimits_{z}^{L}{e}^{-{\alpha }_{q}({\lambda }_{q})({z}^{{\prime} }-z)}{P}_{c}({z}^{{\prime} },{\lambda }_{q})d{z}^{{\prime} }$$

In what follows, we discuss the generation of SpRS photons in the quantum channel through two distinct processes. First of all, we analyze the generation of crosstalk-induced SpRS noise (XT-RS). In this process, the inter-core crosstalk produces a signal at wavelength *λ*_*c*_ of the quantum core, which then produces SpRS at the quantum wavelength *λ*_*q*_ of the same core. In particular, if the quantum signal is propagating in the same direction as the classical signal, the accumulating SpRS power is obtained from Eq. (6) for *z* = *L* where the pump power ${P}_{p}({z}^{{\prime} })$ is the crosstalk power ${P}_{q}({z}^{{\prime} },{\lambda }_{c})$ given by Eq. (13). We denote this case as XT-FRS (with FRS standing for forward SpRS), and the resulting Raman noise power is

16 $$\begin{array}{rcl}{P}_{{\rm{XT}}-{\rm{FRS}}}(L)&=&{P}_{0}{h}_{c,q}({\lambda }_{c}){\eta }_{q}({\lambda }_{c},{\lambda }_{q})\frac{{e}^{-{\alpha }_{q}({\lambda }_{q})L}}{{\alpha }_{c}({\lambda }_{c})-{\alpha }_{q}({\lambda }_{c})}\\ &&\times \left\{\displaystyle\frac{1-{e}^{-[{\alpha }_{q}({\lambda }_{c})-{\alpha }_{q}({\lambda }_{q})]L}}{{\alpha }_{q}({\lambda }_{c})-{\alpha }_{q}({\lambda }_{q})}-\displaystyle\frac{1-{e}^{-[{\alpha }_{c}({\lambda }_{c})-{\alpha }_{q}({\lambda }_{q})]L}}{{\alpha }_{c}({\lambda }_{c})-{\alpha }_{q}({\lambda }_{q})}\right\}\end{array}$$

If, instead, the quantum signal is propagating backward relative to the classical signal, then the Raman noise power is obtained from Eq. (7) for *z* = 0, and the pump power ${P}_{p}({z}^{{\prime} })$ is the crosstalk power ${P}_{q}({z}^{{\prime} },{\lambda }_{c})$ in Eq. (13). We denote this case as XT-BRS (with BRS standing for backward SpRS), and the resulting Raman noise power is

17 $$\begin{array}{rcl}{P}_{{\rm{XT}}-{\rm{BRS}}}(0)&=&{P}_{0}{h}_{c,q}({\lambda }_{c}){\eta }_{q}({\lambda }_{c},{\lambda }_{q})\displaystyle\frac{1}{{\alpha }_{c}({\lambda }_{c})-{\alpha }_{q}({\lambda }_{c})}\\ &&\times \left\{\frac{1-{e}^{-[{\alpha }_{q}({\lambda }_{c})+{\alpha }_{q}({\lambda }_{q})]L}}{{\alpha }_{q}({\lambda }_{c})+{\alpha }_{q}({\lambda }_{q})}-\displaystyle\frac{1-{e}^{-[{\alpha }_{c}({\lambda }_{c})+{\alpha }_{q}({\lambda }_{q})]L}}{{\alpha }_{c}({\lambda }_{c})+{\alpha }_{q}({\lambda }_{q})}\right\}\end{array}$$

Next, we consider the case where SpRS is generated in the classical core by the propagating classical signal and then couples into the quantum core via inter-core crosstalk at the quantum wavelength. We refer to this scenario as RS-XT. In the case of forward-propagating quantum signal, which we refer to as FRS-XT, the Raman noise power is obtained using Eq. (14) for *z* = *L*, where the signal propagating in the classical core ${P}_{c}({z}^{{\prime} },{\lambda }_{q})$ is replaced with ${P}_{{\rm{SpRS}}}({z}^{{\prime} })$ of Eq. (6). Here, the pump signal ${P}_{p}({z}^{{\prime} })$ is given by ${P}_{c}({z}^{{\prime} },{\lambda }_{c})$ of Eq. (12). For the Raman noise power accumulating in the classical core, this procedure yields

18 $${P}_{{\rm{SpRS}}}(z)={P}_{0}{\eta }_{c}({\lambda }_{c},{\lambda }_{q}){e}^{-{\alpha }_{c}({\lambda }_{q})z}\frac{1-{e}^{-[{\alpha }_{c}({\lambda }_{c})-{\alpha }_{c}({\lambda }_{q})]z}}{{\alpha }_{c}({\lambda }_{c})-{\alpha }_{c}({\lambda }_{q})}$$

and for the Raman noise power in the quantum core,

19 $$\begin{array}{lll}{P}_{{\rm{FRS}}-{\rm{XT}}}(L)\,=\,{P}_{0}{\eta }_{c}({\lambda }_{c},{\lambda }_{q}){h}_{c,q}({\lambda }_{q})\frac{{e}^{-{\alpha }_{q}({\lambda }_{q})L}}{{\alpha }_{c}({\lambda }_{c})-{\alpha }_{c}({\lambda }_{q})}\\\qquad\qquad\qquad\times \left\{\frac{1-{e}^{-[{\alpha }_{c}({\lambda }_{q})-{\alpha }_{q}({\lambda }_{q})]L}}{{\alpha }_{c}({\lambda }_{q})-{\alpha }_{q}({\lambda }_{q})}-\frac{1-{e}^{-[{\alpha }_{c}({\lambda }_{c})-{\alpha }_{q}({\lambda }_{q})]L}}{{\alpha }_{c}({\lambda }_{c})-{\alpha }_{q}({\lambda }_{q})}\right\}\end{array}$$

In the case of backward-propagating quantum signal, which we refer to as BRS-XT, the Raman noise power is obtained using Eq. (15) for *z* = 0, where the signal propagating in the classical core ${P}_{c}({z}^{{\prime} },{\lambda }_{q})$ is replaced with ${P}_{{\rm{SpRS}},{\rm{B}}}({z}^{{\prime} })$ of Eq. (7). Here, the pump signal ${P}_{p}({z}^{{\prime} })$ is given again by ${P}_{c}({z}^{{\prime} },{\lambda }_{c})$ of Eq. (12). For the Raman noise power accumulating in the classical core, this procedure yields

20 $${P}_{{\rm{SpRS}},{\rm{B}}}(z)={P}_{0}{\eta }_{c}({\lambda }_{c},{\lambda }_{q})\displaystyle\frac{{e}^{-{\alpha }_{c}({\lambda }_{c})z}-{e}^{-{\alpha }_{c}({\lambda }_{q})z-({\alpha }_{c}({\lambda }_{q})+{\alpha }_{c}({\lambda }_{c}))L}}{{\alpha }_{c}({\lambda }_{c})+{\alpha }_{c}({\lambda }_{q})}$$

and, for the Raman noise power in the quantum core,

21 $$\begin{array}{rcl}{P}_{{\rm{BRS}}-{\rm{XT}}}(0)&=&{P}_{0}{\eta }_{c}({\lambda }_{c},{\lambda }_{q}){h}_{c,q}({\lambda }_{q})\displaystyle\frac{1}{{\alpha }_{c}({\lambda }_{c})+{\alpha }_{c}({\lambda }_{q})}\\ &&\times \left\{\frac{1-{e}^{-[{\alpha }_{c}({\lambda }_{c})+{\alpha }_{q}({\lambda }_{q})]L}}{{\alpha }_{c}({\lambda }_{c})+{\alpha }_{q}({\lambda }_{q})}-\displaystyle\frac{{e}^{-[{\alpha }_{c}({\lambda }_{c})+{\alpha }_{q}({\lambda }_{q})]L}-{e}^{-[{\alpha }_{c}({\lambda }_{c})+{\alpha }_{c}({\lambda }_{q})]L}}{{\alpha }_{c}({\lambda }_{q})-{\alpha }_{q}({\lambda }_{q})}\right\}\end{array}$$

### Impact of IC-SpRS noise on QKD performance

**Fig. 6:**
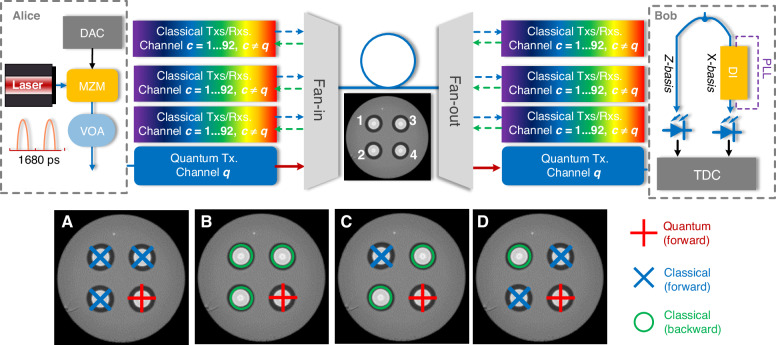
Architecture of co-existence of QKD and DWDM classical signals over MCF. DAC digital-to-analog converter, VOA variable optical attenuator, MZM Mach-Zehnder modulator, TDC time-to-digital converter, DI delay interferometer, PLL phase lock loop. Configurations **A**–**D** represent different integration schemes of QKD and classical communications, differing on the propagation direction of the signals transmitted in the four cores

In this section, we conduct a numerical analysis to explore various configurations for integrating a QKD channel into a fully loaded C-band classical transmission system. These are illustrated in Fig. 6. The fiber parameters employed in this study, including attenuation coefficient, Raman efficiency, and coupling coefficients, were characterized through experimental measurements conducted on the deployed MCFs. A detailed description is provided in the Supplementary Materials. As discussed earlier, we allocate one core for QKD, while the other three cores are dedicated to DWDM classical optical transmission. Specifically, a single-wavelength quantum signal (red + sign) is transmitted in one WDM channel (channel *q*) of core 4, while classical coherent signals (blue crosses or green circles) are transmitted in the other DWDM channels (*λ*_*c*_, *c* = 1–92, *c* ≠ *q*) of the other three cores. These channels range from 191.5 to 196.05 THz (1565.49–1529.16 nm), with 50 GHz spacing, in compliance with the ITU grid standard. The launch power of wavelength-multiplexed classical signals is set to 19.5 dBm, which reduces to 18.3 dBm after the fan-in 1.2 dB insertion loss, corresponding to ~−1.3 dBm per WDM channel in each core, consistent with the experimental configuration presented previously. In this configuration noise photons originating from classical channels can be either out of band, as a result of limited filter insulation or, more importantly, in band as a result of IC-SpRS. We consider the four configurations A, B, C, and D shown in the lower inset of Fig. 6, where a blue × sign represents classical light entering the fiber core, and a green circle represents classical light coming out of the core. For the transmitter, we assume that it generates quantum states at a rate of 595 MHz (1680 ps/state). The receiver with a bandwidth of 0.24 nm is with an additional loss of 2 dB in the Z basis for measurements and 5 dB in the X basis. The InGaAs/InP SPAD model includes a dark count rate of 1500 Hz, a detection efficiency of 0.2, an after-pulsing probability of 0.05, and a hold-off time of 40 ns. Two-time filters with a width of 100 ps are utilized to reduce the impact of dark counts, ensuring the acceptance of at least 50% of the photons for standard detecting systems with a timing jitter below 200 ps.

**Fig. 7:**
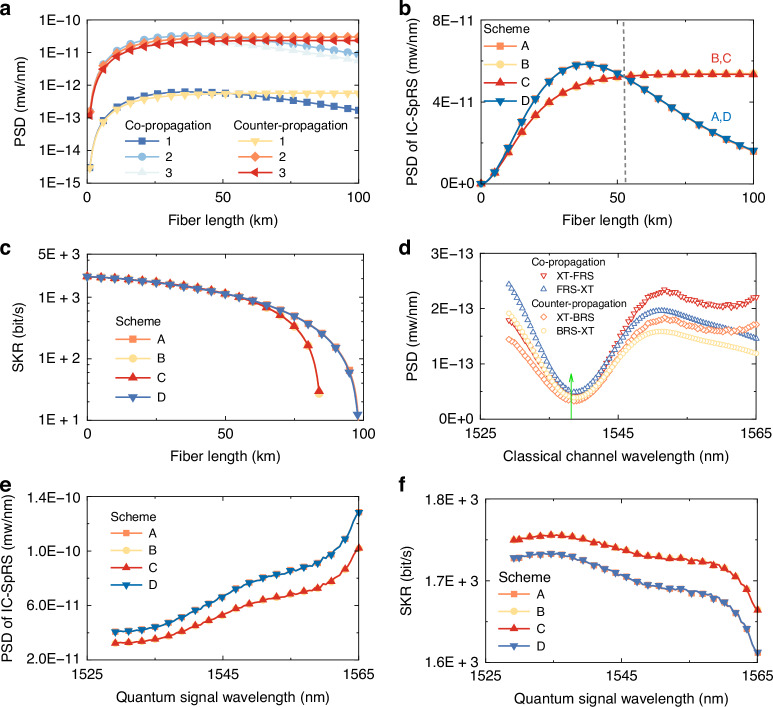
Model-based analysis of SpRS noise and SKR. **a** Power spectral density of forward and backward Raman noise as a function of fiber length. **b** Power spectral density of IC-SpRS noise versus fiber length. **c** Secure key rate as a function of channel attenuation. Quantum signal is always in channel 69 for Fig. (**a**–**c**). **d** Power spectral densities of the Raman noise processes XT-FRS, FRS-XT, XT-BRS, and BRS-XT for a quantum signal in channel 69 (marked by a vertical line), versus the classical channel wavelength. **e** Raman PSD versus the quantum-signal wavelength. **f** Secure key rate versus the quantum-signal wavelength. The fiber length is set to 25.2 km in all cases

First, we fixed the quantum wavelength at channel 69 (1538.19 nm) to be consistent with experiments and investigated the impact of transmission distance on the SpRS noise power at this wavelength. In Fig. 7a, we plot the PSD of the two contributions to IC-SpRS (XT-RS and RS-XT) for each core and transmission direction (co-propagating and counter-propagating) of the classical signal with respect to the quantum signal. The results confirm that the noise from the cores adjacent to the quantum core (cores 2 and 3) is significantly higher than that from the diagonal core (core 1), owing to stronger coupling between adjacent cores. This suggests that the total noise is predominantly influenced by adjacent cores, while classical signals propagating in the diagonal core in either propagation direction have negligible impact on the quantum signal.

In Fig. 7b the PSD of IC-SpRS is plotted as a function of the fiber length for all the transmission schemes illustrated in Fig. 6. Since schemes A and D, as well as schemes B and C differ only in the transmission direction in the diagonal core (whose contribution to the noise is negligible), the curves corresponding to these cases overlap. Most importantly, the figure indicates that counter-propagating interference (schemes A and D) is less noisy than co-propagating interference (schemes B and C) for fiber lengths shorter than ~50 km. Beyond this distance, co-propagation results in lower noise power. Furthermore, the noise in the co-propagating scheme reduces monotonically with distance, whereas the noise in the counter-propagating scheme saturates. This saturation is quite intuitive, as in the counter-propagating scheme most of the noise originates close to the fiber end where the quantum signal is measured. In Fig. 7c we plot the SKR evaluated for the 3-state efficient BB84 protocol versus the fiber length, assuming again a launch power of 19.5 dBm per core for the classical signal. The small advantage that schemes B and C have over schemes A and D for fiber lengths below ~50 km, as observed in Fig. 7b, does not translate into an observable advantage in SKR. However, for fiber lengths greater than ~50 km, schemes A and D, for which the Raman noise reduces with fiber length, outperform schemes B and C, where the Raman noise saturates. The longest fiber length for which a secure key can be extracted is 84 km for schemes B and C, and 98 km for schemes A and D.

Subsequently, we investigated the wavelength dependence of the quantities of interest, having fixed the fiber length to 25.2 km and the classical signal launch power per core to 19.5 dBm. In Fig. 7d, the PSDs of the two processes of XT-RS and RS-XT are plotted for the two schemes of co-propagating and counter-propagating signals. A non-negligible difference between the two processes can be seen in both co- and counter-propagating cases. Such a difference would have not been reproduced by the model in ref. ^32^. In Fig. 7e, the PSD of the Raman noise is plotted versus the quantum signal wavelength for the considered transmission schemes. The results suggest that, from the perspective of Raman noise power reduction, allocating the quantum signal to shorter wavelengths offers a notable advantage. On the other hand, the fiber loss is larger at shorter wavelengths, thereby affecting the quantum signal-to-noise ratio. To take this effect into account, we look again at the performance of the three-state BB84 protocol. The achievable SKR is plotted as a function of the quantum channel wavelength in Fig. 7f. It can be observed that, while allocating shorter wavelengths for the quantum channel is still advantageous, this advantage is only of the order of 6% in terms of SKR. The plot also indicates that schemes B and C outperform schemes A and D across all wavelengths in the presence of Raman noise. Additionally, schemes B and C offer the significant advantage of being unaffected by four-wave mixing, which, unlike SpRS, may become relevant only when classical and quantum signals propagate in the same direction.

**Fig. 8:**
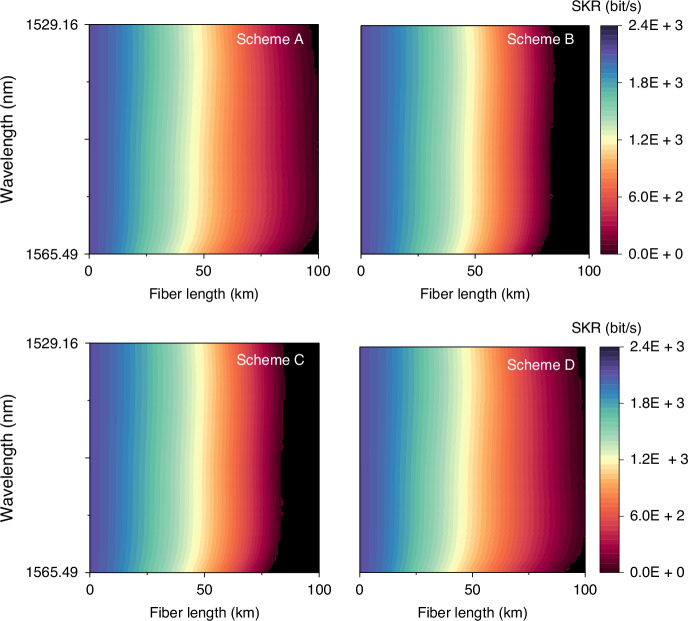
SKR heat maps as a function of fiber length and quantum-signal wavelength for schemes A, B, C, and D

In order to characterize the dependence of QKD performance on fiber length and wavelength simultaneously, we plotted heat maps of SKR for the considered transmission schemes in Fig. 8. A general conclusion drawn from the figure is that, while schemes with counter-propagating classical signals in the neighboring cores (B and C) perform better for fiber lengths below approximately 30 km and the others (A and D) are more effective for longer distances, in both cases the impact of SpRS is minimized in the short-wavelength region.

## Supplementary information

Supplementary material for “Integration of quantum key distribution and high-throughput classical communications in field-deployed multi-core fibers”

## Data Availability

Data and code underlying the results presented in this paper are not publicly available at this time but may be obtained from the authors upon reasonable request.
