# Supplementary material for: Integration of quantum key distribution and high-throughput classical communications in field-deployed multi-core fibers
Source: Light Sci Appl. 2025 Aug 13;14:274. doi: 10.1038/s41377-025-01982-z (PMC12350615; doi:10.1038/s41377-025-01982-z)
Supplement: Supplementary file 1 — Supplementary material for “Integration of quantum key distribution and high-throughput classical communications in field-deployed multi-core fibers” [file 41377_2025_1982_MOESM1_ESM.pdf]

Supplementary material for "Integration of quantum key distribution and high-throughput classical communications in field-deployed multi-core fibers"

Qi Wu[1,2,3]†, *Domenico Ribezzo*[1,4]†, *Giammarco Di Sciullo*[1], *Sebastiano Cocchi*[4], *Divya Ann Shaji*[1], *Lucas Alves Zischler*[1], *Ruben Luis*[5], *Paolo Serena*[6,7], *Chiara Lasagni*[6,7], *Alberto Bononi*[6,7], *Tetsuya Hayashi*[8], *Alessandro Gagliano*[9], *Paola Martelli*[9], *Alberto Gatto*[9], *Paolo Parolari*[9], *Pierpaolo Boffi*[9], *Davide Bacco*[4], *Alessandro Zavatta*[10], *Yixiao Zhu*[3], *Wweisheng Hu*[3], *Zhaopeng Xu*[11], *Mark Shtaif*[12], *Andrea Marotta*[1,7], *Fabio Graziosi*[1,7], *Antonio Mecozzi*[1,7], *Cristian Antonelli*[1,7]

† These authors contributed equally to this work.

Corresponding authors: qi.wu@student.univaq.it; cristian.antonelli@univaq.it

[1] Department of Physical and Chemical Sciences, University of L'Aquila, L'Aquila 67100, Italy

[2] Photonics Research Institute, Department of Electrical and Electronic Engineering, The Hong Kong Polytechnic University, Hong Kong, China

[3] Department of Electronic Engineering, Shanghai Jiao Tong University, Shanghai 200240, China

[4] Department of Physics and Astronomy, University of Florence, Via Sansone 1, Firenze 50019, Italy

[5] Photonic System Laboratory, NICT, Koganei, Tokyo 184-0015, Japan

[6] Department of Engineering and Architecture, Università degli Studi di Parma, Parma 43124, Italy

[7] CNIT National Laboratory of Advanced Optical Fibers for Photonics, L'Aquila 67100, Italy

[8] Sumitomo Electric Industries, Ltd., 1, Taya-cho, Sakae-ku, Yokohama, Kanagawa 244-8588, Japan

[9] Department of Electronics, Information and Bioengineering, Politecnico di Milano, Milano 20133, Italy

[10] National Institute of Optics (CNR-INO), Largo E. Fermi 6, Firenze 50125, Italy

[11] Pengcheng Laboratory, Shenzhen 518055, China

[12] Department of Physical Electronics, Tel Aviv University, Tel Aviv 69978, Israel

Field-deployed multi-core fiber characterization and results

To comprehensively analyze the impact of IC-SpRS noise, we experimentally characterized the wavelength-dependent fiber parameters involved in the model in our deployed multi-core fiber. This is an uncoupled-core four-core fiber deployed in L'Aquila, Italy. While the detailed fiber-design characteristics can be found in [43], here it is relevant to remind that the channel is made by four fiber strands of 6.3 km each housed in the same loose tube, which can be concatenated to form a link of 25.2 km, corresponding to four laps around the city downtown. The experimental setup for this measurement is illustrated in Fig. S1. By sending C-band amplified spontaneous emission (ASE) from an Erbium-Doped fiber amplifier (EDFA), we measured the attenuation for each core and the coupling coefficients for each pair of cores,

while a laser was employed to characterize the Raman efficiency for each core, where an optical processor (OP) was used to suppress the laser side modes. The fiber loss coefficient of each core, plotted as a function of wavelength in Fig. S2(a), reduces with increasing wavelength, as expected from the wavelength dependence of Rayleigh scattering. To characterize the Raman efficiency of our deployed MCF, we used the experimental setup described in [17], with a pair of fan-in fan-out (FIFO) devices providing access to the individual cores. The frequency-dependent SpRS efficiency was derived from its relation to the measured attenuation and the SpRS noise power [17]. To estimate the equivalent Raman efficiency for an arbitrary pump-light wavelength, we proceeded as detailed in [S1]. That is, given the efficiency measured at λ_q for a pump at λ_c, which in our formalism is denoted by $\eta(\lambda_c, \lambda_q)$, the efficiency at λ_q' for a pump at λ_c' (with $\lambda_c^{-1} - \lambda_q^{-1} = \lambda_{c'}^{-1} - \lambda_{q'}^{-1}$) is obtained as

$$\eta(\lambda_{c'}, \lambda_{q'}) = \left(\frac{\lambda_q}{\lambda_{q'}}\right)^4 \eta(\lambda_c, \lambda_q) \tag{S1}$$

Since the measurement of the Raman efficiency requires knowledge of fiber loss, which in our case was only available in the C-band, our experiment was performed in two stages. First, we used a pump at 1525 nm to measure the Raman efficiency at positive wavelength shifts, from 1525 nm to 1565 nm, then we used a pump at 1567 nm to measure the Raman efficiency at negative wavelength shifts, from 1567 nm to 1527 nm. This allowed us to estimate the Raman efficiency for any pair of wavelengths within the entire C-Band. For illustration, the efficiency curve resulting from Eq. (S1) for a pump light at 1550 nm is plotted in Fig. S2(b). The plot shows that the Raman efficiency at wavelengths beyond the pump is higher than at wavelengths below the pump, aligning with the established understanding that SpRS is more efficient at positive wavelength shifts [S1]. The curves also show that the two peaks are approximately ±13 nm from the pump wavelength, as highlighted in Fig. S2(b), which is consistent with measurements in single-mode fibers [17]. The coupling coefficients $h_{c,q}$, which characterize the crosstalk, were determined as the ratio of the output power of the unexcited core c to the input power of core q normalized to the fiber length, and of course to the attenuation of core q[1]. Figure S2(c) shows the measured coupling coefficients, ranging from -60 dB·km^{-1} to -55 dB·km^{-1} for adjacent cores, and dropping below -70 dB·km^{-1} for diagonal cores. The heat map in Fig. S2(d) represents the time-and core-averaged coupling coefficients. It clearly shows that coupling between adjacent cores is significantly higher than that between diagonal cores. This observation suggests that the influence of diagonal cores is not a dominant factor in IC-SpRS.

Based on the measurements described above, in Fig. S2(e) we investigated the evolution of the PSD of IC-SpRS noise as a function of the fiber length with the setup shown in Fig. 5(a), where the pump is set at 1550 nm. The figure makes use of the data measured for cores 3 and 4. The four panels, from left to right, correspond to XT-FRS, FRS-XT, XT-BRS, and BRS-XT, respectively. It can be observed that the noise generated at wavelengths above the pump is stronger than that at negative wavelength shifts. Additionally, the forward Raman noise initially increases with the increasing fiber length, but it decreases once the fiber length exceeds a certain threshold, which is determined by the inherent attenuation of the fiber cores.

---

[1] This can be seen by expanding Eq. (13) to first order with respect to $(\alpha_c - \alpha_q)z$, which yields $P_q(z, \lambda_c) = h_{c,q}(\lambda_c) z P_0 e^{-\alpha_q z}$.

In contrast, the backward Raman noise increases with the increasing fiber length and then saturates. These observations – consistent with [32] – suggest that for long-distance transmission, co-propagation of classical and quantum signals is preferable to minimize the impact of SpRS noise.

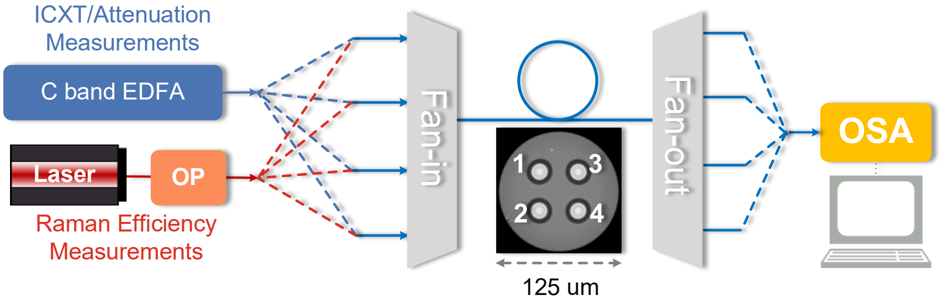

Fig. S1 Experimental setup for the MCF characterization. OP: Optical processor; EDFA: Erbium-doped fiber amplifier; OSA: Optical spectrum analyzer.

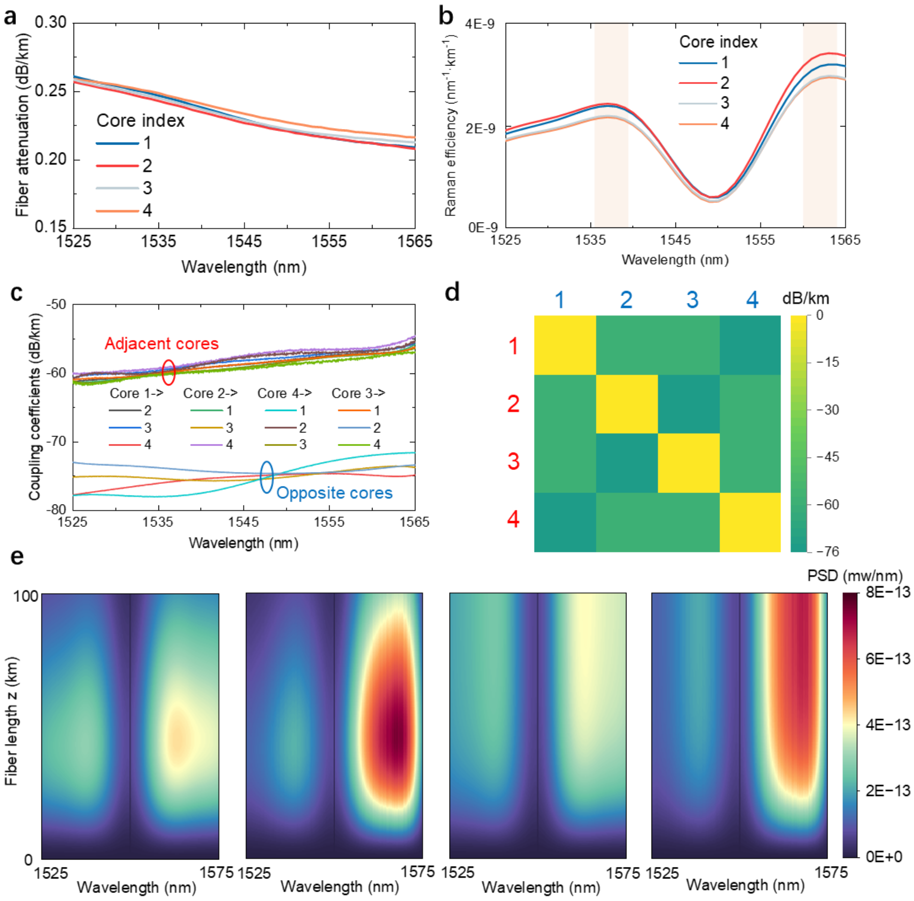

Fig. S2 (a) Fiber-core attenuation as a function of wavelength. (b) Raman efficiency versus wave- length for a pump light at 1550 nm. (c) Inter-core coupling coefficient as a function of wavelength. (d) Average coupling coefficient across cores and wavelengths. (e) PSD heat maps of the four SpRS noise generation processes: XT-FRS, FRS-XT, XT-BRS, and BRS-XT (from left to right), for a pump at 1550 nm.

References

[S1] Bahrani S, Razavi M, Salehi J A. Wavelength assignment in hybrid quantum-classical networks. Scientific

reports, **8**, 3456 (2018).
